# Clinically relevant aspects of professional follow-up care for implant patients

**DOI:** 10.3389/fdmed.2025.1565151

**Published:** 2025-05-09

**Authors:** Philipp Sahrmann, Catherine Giannopoulou

**Affiliations:** ^1^ Department of Periodontology, Endodontology and Cariology, University Center for Dental Medicine Basel UZB, University of Basel, Basel, Switzerland; ^2^ Division of Regenerative Dental Medicine and Periodontology, University Clinics of Dental Medicine (CUMD), University of Geneva, Geneva, Switzerland

**Keywords:** peri-implant diseases, prevention, maintenance, peri-implantitis, dental implants

## Abstract

The detailed 2023 guidelines for the prevention and treatment of peri-implantitis describe evidence-based measures for the professional care and prevention in patients with dental implants. However, there remains a lack of reliable data from randomized clinical trials on many critical points, particularly regarding specific diagnostic steps and treatment decisions, to provide definitive guidance and protocols for implementation during recall sessions in daily practice. This narrative review seeks to address this gap by highlighting the critical aspects of follow-up care that should be monitored during periodontal maintenance to ensure the health of peri-implant tissues and enable timely intervention when peri-implant health is compromised.

## Introduction—Evidence-based guidelines

The clinical guidelines of the European Association of Periodontology, published in March 2023, provide detailed recommendations for a scientifically supported clinical approach based on a total of 13 systematic reviews (1). These reviews examined the published clinical evidence regarding the health of peri-implant tissues as well as the prevention and treatment of related diseases. One might therefore assume that further specialist contributions on pre- and post-treatment care would be rendered unnecessary by reading the corresponding guidelines. Unfortunately, many clinically relevant questions have not yet been sufficiently studied so far to provide reliable answers. In light of the potential negative effects of additional measures—where even increased costs are considered a drawback—the recommendations for many seemingly plausible approaches (like for example the use of powder abrasive devices, topically administered disinfectants and the use of bone graft materials, to name just some of them) are often negative. As a result, the treatment strategies that are effectively recommended appear to be extremely conservative. However, the limitation of lacking clinical evidence creates an opportunity to focus on those therapeutic steps, that have been fundamentally studied and well-understood, and to implement them meticulously and effectively in clinical practice. This allows the limited time available during follow-up sessions to be used as effectively as possible for the maximum benefit of the patient.

Specific recommendations especially for peri-implant tissues are particularly important for several reasons. First, the progression of irreversible tissue loss around implants occurs relatively quickly, making early intervention crucial (2). Second, titanium dioxide, which remains the most widely used implant material, is very soft and prone to significant changes in surface morphology during mechanical processing, posing potential challenges for long-term durability (3, 4). Third, the peri-implant environment is typically characterized by the necessity of prosthetic restorations, which often restrict access for effective mechanical cleaning, further complicating maintenance efforts (5, 6).

When it comes to the development of peri-implant inflammation, it is important to distinguish between the biofilm, which acts as the essential prerequisite (“sufficient component cause”) for the immune response (7–10), and the risk factors that significantly influence the progression rate of tissue destruction (7). Understanding these dynamics is critical for guiding effective preventive and therapeutic strategies.

The presence of biofilm-specific antibodies, such as pathogen-associated molecular patterns (PAMPs) and lipopolysaccharides, triggers the cascade of the immune response: Chemokine-mediated lymphocytes, macrophages, and polymorphonuclear neutrophil granulocytes are attracted and activated through resident keratinocytes and patrolling white blood cells. In the local tissue, under the influence of prostaglandin E, extravasation and edema formation occur, while enzymes like MMP-8 degrade collagen I and III at the peri-implant interface with the bone (8, 9). This cascade can be easily and significantly influenced by minimizing the exposure of host tissue to biofilm.

Among the many known risk factors for peri-implantitis (7, 10, 11), most—such as genetic predisposition (often clinically manifested by periodontal breakdown in remaining periodontium), implant location/position, implant design (bone or soft tissue level philosophy), and connection elements—can hardly be influenced once follow-up begins or problems arise. However, a few factors, such as oral hygiene, smoking habits, and some general medical conditions (12), can be corrected.

According to the guideline, the three main tasks in the periodically repeated recall session are professional dental cleaning, effective instruction for individualized oral hygiene, and, if necessary, tailored smoking cessation counseling and a thorough check of overall health (1). This can be quite an ambitious program for a session typically scheduled between 30 and 50 min.

## Diagnosis and triage

Therefore, it is important to categorically exclude therapeutic steps that are not comprehensively performed during maintenance: While healthy peri-implant conditions as well as peri-implant mucositis—characterized by bleeding at more than one of six sites per implant, with stable probing depths and radiologically determined bone level (13)—are core components of the prophylaxis session, as is the secure diagnosis of peri-implantitis (suppuration, increasing probing depths, and progressive marginal bone loss), the targeted treatment of manifest peri-implantitis often exceeds the scope of the classic recall. It requires a separate appointment with systematically sequenced treatment steps, which often include surgical procedures and are therefore reserved for the specialized dentist (1).

**Figure 1:**
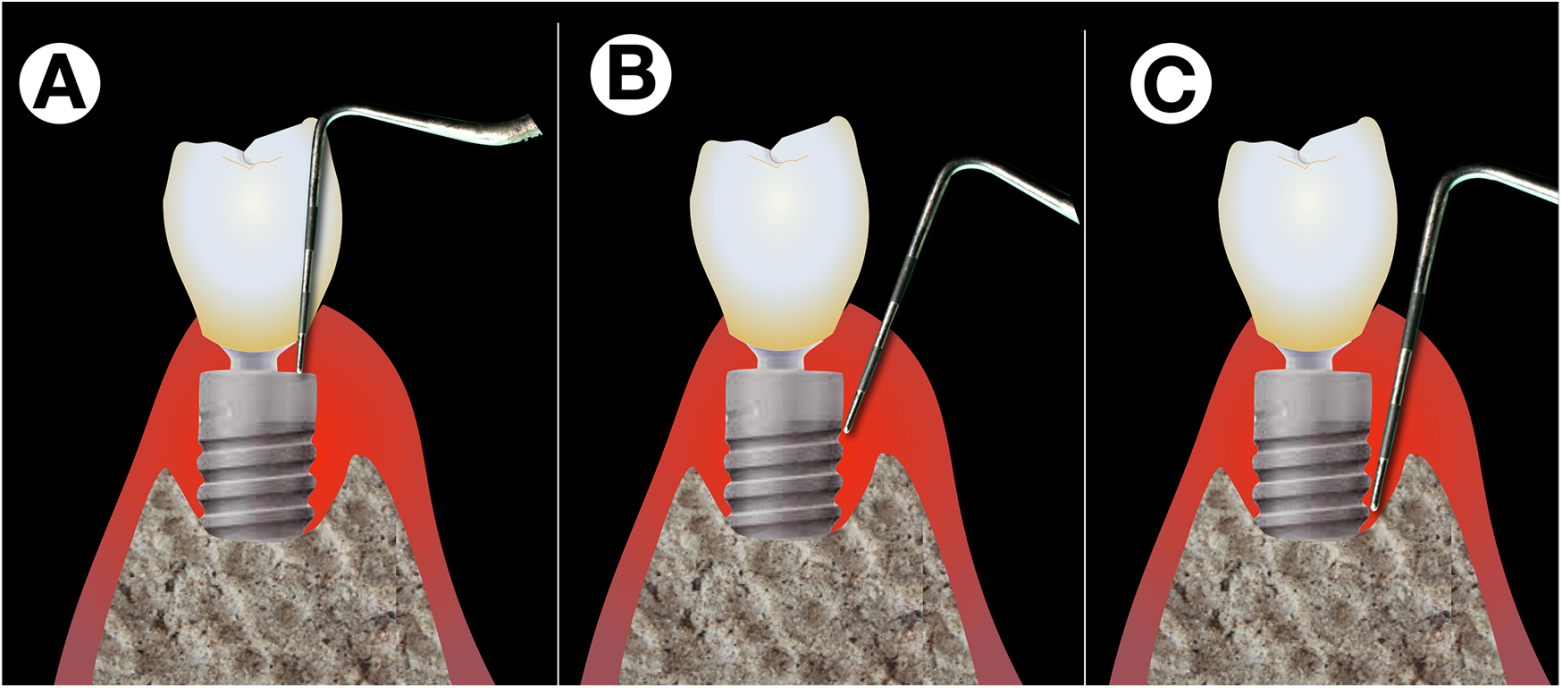
Difficulties with peri-implant probing. Exact peri-implant probing is difficult on different levels: While the shape of the implant's shoulder and platform switching renders probing difficult in the upper part **(A)**, correct probing is difficult due to pain sensations of the patient while the operator tries to verify whether the tip of the probe is still stuck in the implants’ threads **(B)** or has already reached the most coronal point osseo-integrated part **(C)**.

The precise diagnosis of peri-implantitis is considerably more complicated than that of periodontitis (14), partly due to the morphology in the collar area of implants, which lies within the emergence zone from the surrounding soft tissue (Figure 1). Implants are generally narrower in diameter than corresponding teeth. Thus, to allow for an aesthetic appearance, a strongly funnel-shaped widening of the crown and implant complex within the supracrestal soft tissue is required. This significantly complicates access for peri-implant probing as a standard diagnostic tool, especially since peri-implant tissue in an inflammatory state is highly sensitive to pain.

Two easy measures can significantly simplify the diagnostic process: On the one hand, a mild infiltration anesthesia (requiring only a small amount of anesthetic) enables precise and reliable probing without the practitioner being influenced by the patient's pain. Peri-implant probing should first be performed and documented at the timepoint of insertion of the restoration to facilitate the secure and early detection of deepening pockets (1). While probing, a careful analysis of the vertical implant position with regard to the bone margin helps to control the correct measurement. On the other hand, gently stroking the peri-implant soft tissue from apical to coronal under light pressure with the fingertip of the practitioner allows for an accurate analysis of any possible peri-implant exudate. It is important to first dry the sulcus under an air stream and then carefully observe any fluid exuding from the sulcus during the stroking process. The absence of exudate suggests peri-implant health, while clear mucosal crevicular fluid indicates a serous inflammation (peri-implant mucositis). Not infrequently, a drop of cloudy exudate or pus unexpectedly emerges from a region that was barely noticeable during cursory probing, which is a clear sign of peri-implantitis and necessitates further diagnostic steps (discussed probing under anesthesia, new radiograph). Regarding sensitivity and specificity of this finger stroking technique, future studies have yet to provide precise data.

**Figure 2:**
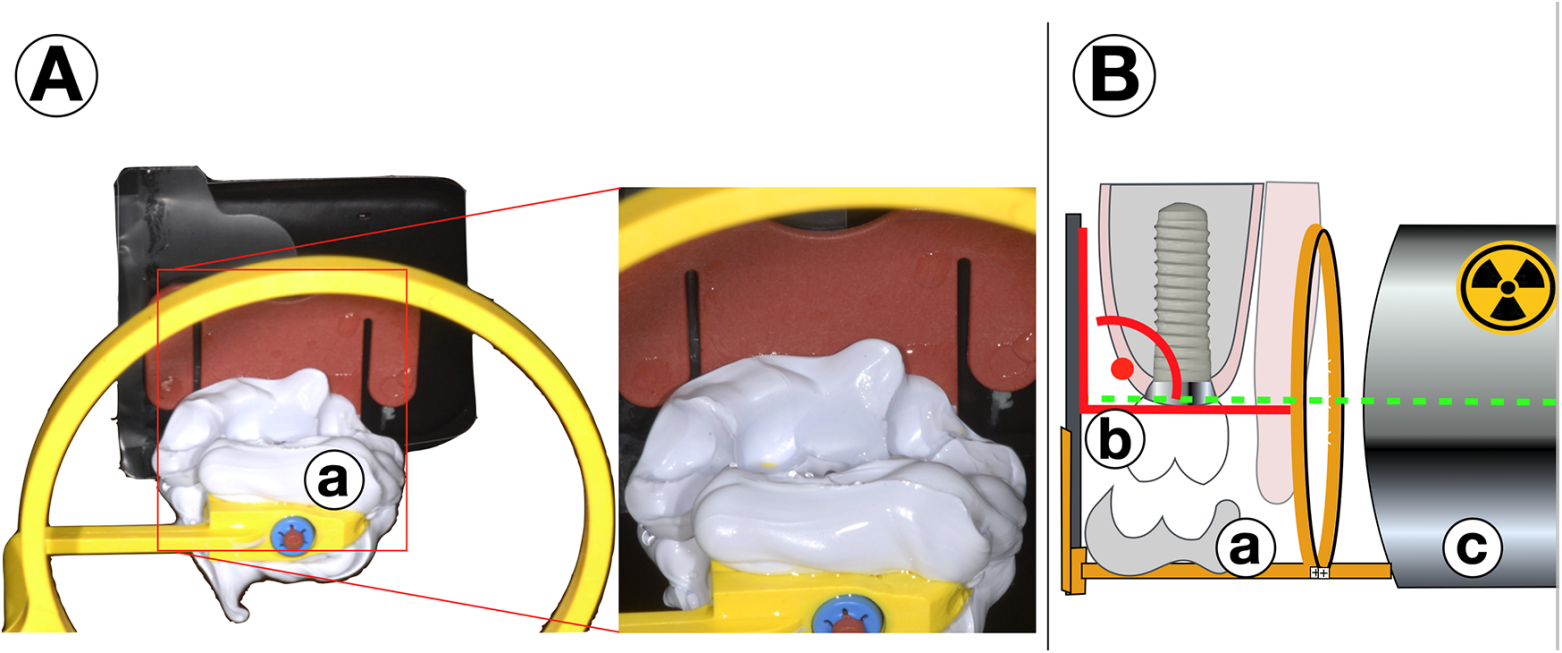
**(A)** Standardized radiographs with individualized stents. After insertion of the prosthetical parts, for the baseline radiograph the occlusion is registered by a quick-setting a-silicone on the occlusion bar of the stent (a) The material remains fixed to the x-ray tray. For every follow-up radiograph **(B)** the bite (a) is positioned exactly on teeth and restoration (b) and a right-angle image is done with the same settings and from the same direction in order to produce perfectly comparable pictures (c).

Regarding an early and highly sensitive analysis of potential marginal bone loss, the standardization of single-tooth radiographs using a simple individual stent is recommended. For this, after the insertion of the prosthetic restoration, the occlusion is imprinted using a quick-setting A-silicone material in the bite area of the conventional radiographic holder during the baseline radiograph, as recommended by the guidelines. The bite registration is then left on the radiographic stent. This individualized stent can be reused during each follow-up examination, ensuring that the direction of each subsequent radiograph perfectly matches that of the baseline examination (Figure 2) (15). For full arch restorations the areas depicted on one radiograph should be chosen diligently for the first documentation in order to make the best compromise of exact display of each single implant and the number of radiographs needed. While the fabrication of such a stent is easily and quickly done, storage and continuous use might be a challenge for a practice with a high proportion of implant cases. Stents might then be fabricated for patients and implants at a higher risk for failure or more complex restorations.

## Optimization of oral hygiene

The guidelines are clear in their demand for optimized oral hygiene as a prerequisite for peri-implant health and the prevention of inflammation (1). It is more than just a cosmetic issue that, on the one hand, the corresponding recommendations are based solely on indirect evidence (there is, in fact, no clinical study on this), and on the other hand, it is explicitly stated that there is no data to recommend a “best brushing technique” as such.

**Figure 3:**
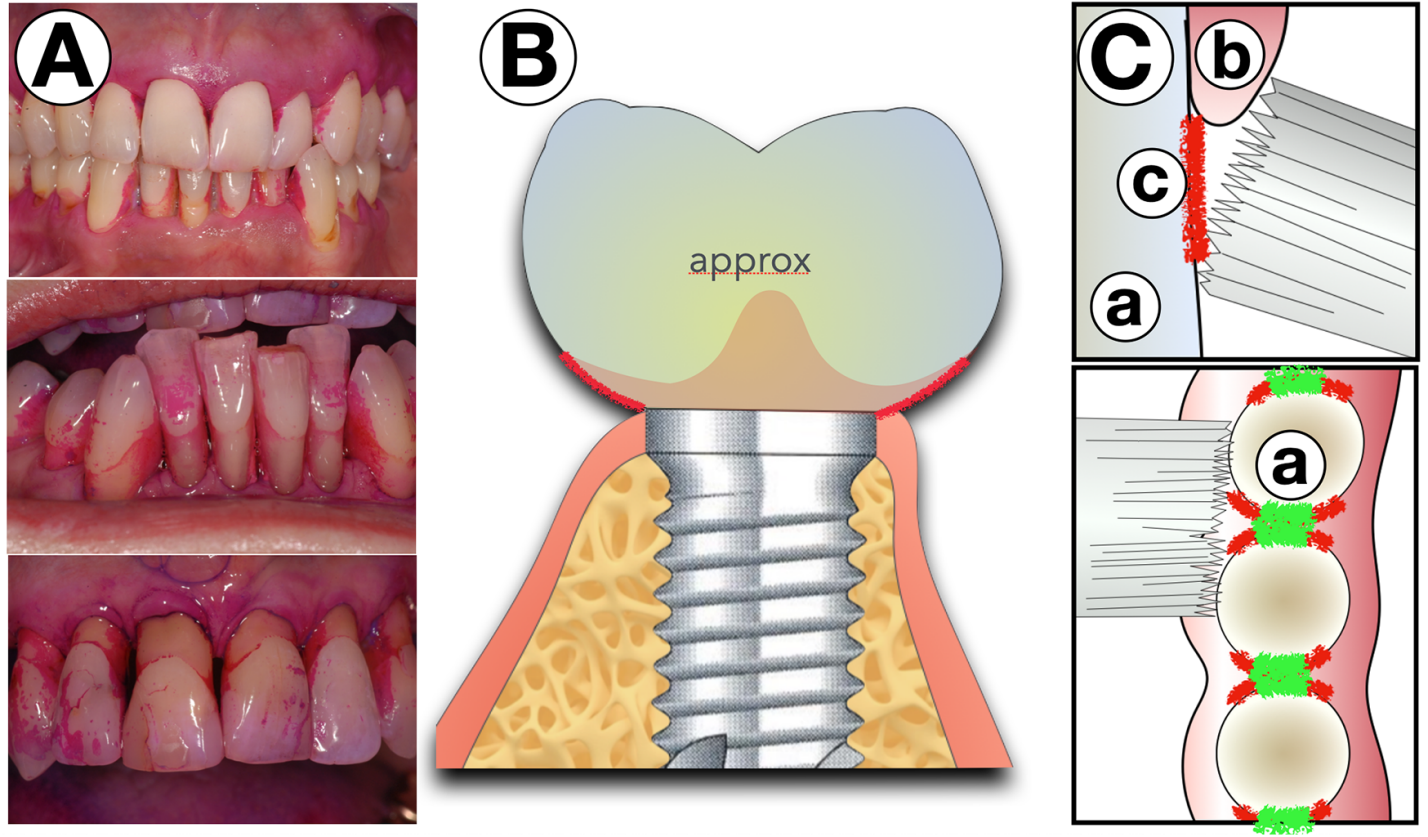
Areas with residual plaque after conventional brushing. **(A)** Areas which typically remain uncleaned by conventional brushing revealed by plaque revelator. **(B)** Sketch that displays these areas from approximally. **(C)** On the prostetical parts (a) closed to the margo mucosae **(b)** the even bristle field of a conventional brush cannot enter the residually uncleaned surfaces (c) The green areas may be brushed well by a brush.

**Figure 4:**
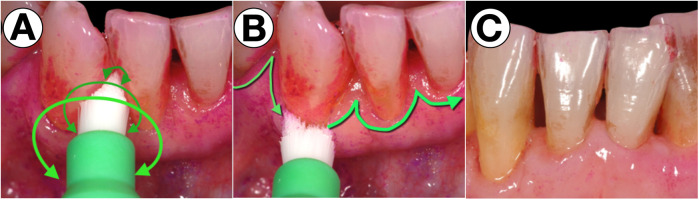
Specialized sonic-activated brush head for the residually uncleaned areas. With a small, round and pointed brush head, which is ideally activated sonically, the problematic areas are easy to clean by rotating movements **(A)** and movements along the margo mucosa with a bristle angulation of 45° against the sulcus **(B)** to efficiently remove biofilms from the respective areas **(C)**.

While the reasons for these limitations—ethical considerations and inter-individual differences in the morphology of the gums and implant restoration—are evident, the lack of scientific evidence is not necessarily a true limitation: after all, the Latin term *evidentia* means nothing more than “clarity” or “illustration.” The illustrative display of plaque distribution, as it can be done precisely in just a few seconds using commercially available plaque revelators, can be clinically performed quickly and easily, serving as a significant aid in patient motivation. The problematic areas are almost always the area immediately adjacent to the mucosal margin of the implant crown restoration (Figure 3). The gum line in implants often does not smoothly transition to the prosthetic reconstruction but forms an accentuated step at the margin, which is not efficiently reachable with conventional toothbrushes (Figure 4). Another problematic area is the interdental space. Since the implant diameters are normally shallower than those of teeth, the interdental/inter-implant areas are considerably wider than those of the natural dentition. These spaces can perfectly be addressed by the use of interdental brushes of various sizes. While the narrowest part can be sufficiently cleaned with interdental brushes (IDB), the remaining areas between the line angles (interdental funnels) are left nearly uncleaned.

The authors’ observations in daily practice indicate that these areas can be effectively cleaned using small, round, and tapered brush heads. When activated by sound, these brushes replicate the manual, difficult-to-perform shaking motion of the Bass technique, making them a highly efficient tool for optimized oral hygiene, as illustrated in Figure 4.

It is important to note that, to date, no scientific studies have provided evidence supporting the superiority of any specific brushing technique around implants. Therefore, practitioners must rely on existing clinical evidence.

**Figure 5:**
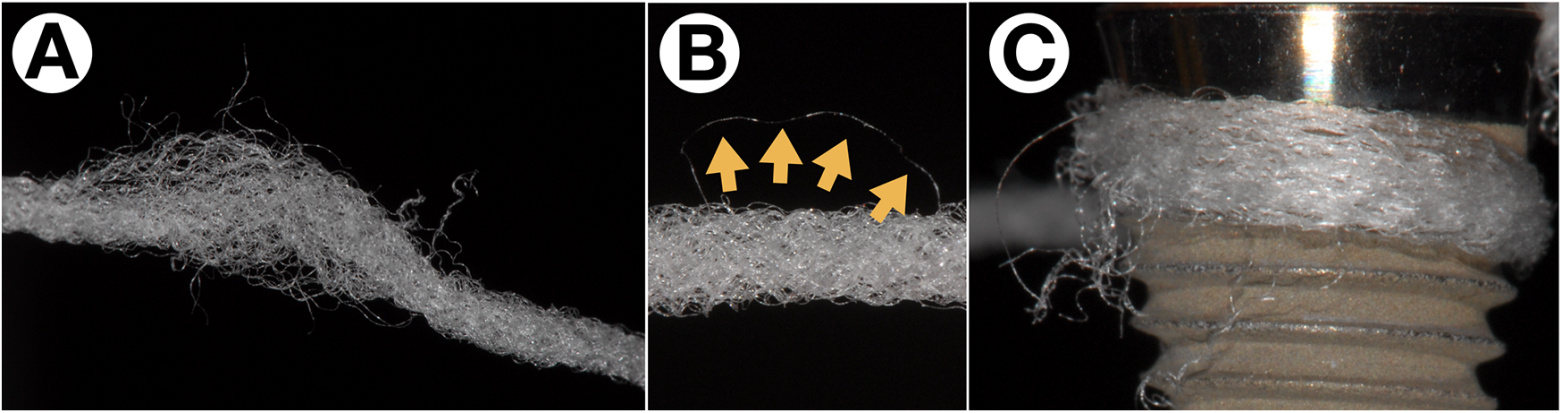
Fiber retention from “superfloss” products. Especially fluffy floss types **(A)** are prone to lose single fibers **(B**, yellow arrows**)** especially on rough implant surfaces **(C)** or incongruencies between implant and prosthetical parts.

While dental floss appears suitable for areas just below the mucosal margin, there is a particular risk with fluffy “superfloss” products. Rough implant surfaces or inconsistencies with the prosthetic components can pull small fibers from the floss texture, which remain in the sulcus and become colonized by biofilm, leading to a rapid, severe mucosal inflammation within days (Figure 5). In a retrospective study on peri-implantitis patients with an intensive oral hygiene protocol even submucosally remnants of floss fibers could be detected. On removal, in 90% of the cases a significant improvement of the peri-implant inflammation was observed (16). Although removing such filaments leads to a quick remission of inflammation, the use of dental floss should not be generalized. A specific product and its individual usage should be recommended and instructed based on the patient's needs.

With perfect peri-implant conditions and perfectly fitting restorations, however, the use of dental floss seems not to be a problem, and the benefits will prevail potential risks (17).

In implant-supported bridges the pontic area may be cleaned well with floss products with stiff ends which facilitate insertion. Even though biofilm should be fought in the whole dentition—be considered less critical than the areas where the implants actually emerge—pontics make a tight contact to most often keratinized soft tissue and a fully epithelialized surface, and not to a sulcular area which is highly responsive to the presence of biofilms.

## Debridement in practice

For professional biofilm removal in the practice setting, the guidelines primarily recommend mechanical tools and advise against the use of pharmacological agents such as antibiotics or antiseptics. Which of the tools, ranging from ultrasound-activated plastic tips to chitosan brushes or powder spray devices with low-abrasive media, is the most suitable remains unclear based on randomized clinical studies, and thus no recommendation is made. However, laboratory studies show that the mechanical treatment of implant surfaces is problematic for two relevant reasons: First, instruments made of relatively hard materials, such as steel or titanium, will alter the surface morphology of implants (18, 19). Essentially, this means that moderately rough surface areas are smoothed out during treatment. While this may reduce the biocompatibility of these areas, which would be disadvantageous for desired osseointegration, it also lowers the susceptibility to biofilm accumulation (20). This is a clear advantage once the corresponding areas remain exposed to the oral environment and need to be cleaned by the patient. On the other hand, machined or polished surface areas are roughened by such instruments made of hard materials, which is highly disadvantageous in the sensitive area of the emergence profile due to an enhanced adherence of biofilm (19, 21, 22).

When softer materials such as plastic and carbon are used in cleaning instruments, it must be assumed that small particles from the material will be abraded and left on the surfaces that are actually being cleaned (19). These particles can in turn quickly accumulate biofilm, which calls into question their effectiveness for cleaning purposes. A middle ground between surface changes and abrasion on the implant surfaces is represented by the plastic PEEK: It changes the surface morphology less significantly than steel or titanium but still leaves particles on the titanium substrate when used (19). *In-vitro* studies using low-abrasive powders like glycine or erythritol, on the other hand, show superior cleaning efficacy compared to other mechanical methods, without visibly altering the surface morphology (18, 23). However, only a few clinical studies to date show advantages with their use (24, 25). Other adjunctive to mechanical instrumentation methods that have been considered, include photodynamic therapy, laser treatment and the use of probiotics. Again, there is no conclusive evidence, as no study could show significant benefits of one of these methods, beyond what is achieved with the mechanical instrumentation alone (26).

## Recall interval

While the importance of well-functioning maintenance in periodontology has long been known (27–29), a recent prospective clinical study in a private practice setting impressively demonstrated the high benefit of a strictly managed recall program with good compliance in terms of the incidence of peri-implantitis (30). While, with good compliance, the incidence of inflammation in the remaining dentition was only 33% over 20 years, with virtually no inflammation, all patients with poor compliance exhibited peri-implant inflammation over the same period.

Regarding recall frequency, the guidelines also provide no clear guidance. Several studies that address this question identify a range of parameters that modify the time between recall appointments. In addition to risk factors such as general health (diabetes, smoking), increasing probing depths, the maintainability of the implant restoration, and the most sensitive of these parameters, bleeding on probing (BoP), are mentioned (9, 31, 32). A simplistically, almost trivial but practical rule of thumb could be: As long as the remaining dentition and implants do not show an increased tendency for inflammation, the recall interval is adequate. If more sites with BoP appear, the interval should be shortened accordingly to address inflammation at the stage of peri-implant mucositis. From a practical standpoint, it seems of course both safer and easier to begin with short intervals of up to three months and gradually extend them to determine the optimal frequency for each individual patient. Intervals of up to 12 months have been reported to remain sufficient if potential risk factors are well controlled (30).

## Summary

Given the relatively rapid progression of peri-implant tissue degradation, secure and sensitive diagnostics are of great importance. Simple additional measures, such as stroking of the vestibular gums in apico-coronal direction to detect sulcus fluid or pus and probing under anesthesia, help to overcome the implant-specific challenges in diagnostics. Oral hygiene, as a central and timely means of disrupting biofilm development, requires significant discipline and consistency from the patient. Therefore, it is crucial that truly efficient tools are instructed for the clinically relevant areas. A small, round, and pointed sonic-driven brush head represents such a useful aid. In professional cleaning, in addition to quick and as comprehensive as possible cleaning, minimizing potential damage through instrumentation is key. In this regard, powder abrasive devices with low-abrasive media show great promise based on *in vitro* studies, although superior results in clinical studies are still largely lacking. When determining the recall interval, bleeding on probing (BoP) should be used as the most sensitive parameter to detect peri-implant inflammation early, while it is still largely manageable.
